# PPARG Polymorphisms Are Associated with Unexplained Mild Vision Loss in Patients with Type 2 Diabetes Mellitus

**DOI:** 10.1155/2019/5284867

**Published:** 2019-12-12

**Authors:** Tao Li, Xian Xu, Yi Xu, Peiyao Jin, Jianhua Chen, Yongyong Shi, Haidong Zou

**Affiliations:** ^1^Department of Ophthalmology, Shanghai General Hospital, Shanghai Jiaotong University, Shanghai, China; ^2^Shanghai Eye Diseases Prevention & Treatment Center, Shanghai Eye Hospital, Shanghai, China; ^3^Shanghai Key Laboratory of Ocular Fundus Diseases, Shanghai, China; ^4^Bio-X Institutes, Key Laboratory for the Genetics of Developmental and Neuropsychiatric Disorders (Ministry of Education), The Collaborative Innovation Center for Brain Science, Shanghai Jiaotong University, Shanghai, China; ^5^Shanghai Key Laboratory of Psychotic Disorders, Shanghai Mental Health Center, Shanghai Jiaotong University School of Medicine, Shanghai, China

## Abstract

**Objectives:**

To investigate whether the presence of peroxisome proliferator-activated receptor gamma (PPARG) gene polymorphisms is associated with unexplained mild visual impairment (UMVI) in patients with type 2 diabetes mellitus (T2DM).

**Methods:**

A total of 135 T2DM residents with UMVI and 133 with normal vision (NV; best-corrected visual acuity ≥ 20/25 in both eyes) were enrolled. UMVI was defined as best-corrected visual acuity (BCVA) < 20/25 and ≥ 20/63 in both eyes, with no visual impairment-causing diseases found. Four PPARG gene single-nucleotide polymorphisms (SNPs) (rs3856806, rs1801282, rs709158, and rs10865710) were assessed with the HAPLOVIEW 4.0 software to examine the statistical association of PPARG polymorphisms and UMVI in patients with T2DM.

**Results:**

Four SNPs qualified the Hardy–Weinberg equilibrium (*p* > 0.05). The frequency of genotype GC at SNP rs10865710 was significantly higher in the UMVI group than in the NV group (*p* < 0.001; GG + GC versus CC) (OR = 8.94, 95% CI: 4.90–16.31), whereas genotype CC decreased the risk (OR = 0.07, 95% CI: 0.03–0.14). Genotype TT at SNP rs3856806 was strongly associated with UMVI (*p* < 0.0001, TT + TC versus CC) (OR = 4.74, 95% CI: 2.68–8.54), whereas genotype CC appeared to be protective for UMVI (OR = 0.55, 95% CI: 0.37–0.82).

**Conclusions:**

Susceptibilities of PPARG variants may lead to differences in PPARG transcription, result in early function loss of retinal photoreceptor cells, and eventually cause UMVI.

## 1. Introduction

Diabetes is a group of metabolic disorders characterized by hyperglycemia resulting from defects in insulin secretion, insulin action, or both [1, 2]. The World Health Organization (WHO) estimated that by 2025, there would be 300 million people worldwide with diabetes mellitus [3], and Type 2 diabetes mellitus (T2DM) accounted for 90%–95% of those with diabetes [1]. Over the last three decades, there had been a major rise in the prevalence of T2DM globally [4]. T2DM is associated with many complications, among which ocular complications are common and usually emerging earlier than other complications [5]. Owing to complications such as cataracts, diabetic retinopathy (DR), and glaucoma, the prevalence of visual impairment is much higher in the T2DM population than the nondiabetic populations. Over the past decades, measures for prevention of visual impairment focused on moderate to severe visual impairment and blindness (best-corrected visual acuity (BCVA) < 20/63) [6]. Accordingly, few health administration members or ophthalmologists paid attention to mild visual impairment (BCVA < 20/25 and ≥20/63), [6] which also reduced the activities of daily living and life quality of patients with T2DM [7, 8].

From October 2014 to January 2015, we conducted a cross-sectional, epidemiological study of eye disease among 2,216 adults with T2DM in the Xinjing community, Shanghai [9]. Mild visual impairment was found in 1,891 eyes—42.7% of all eyes examined (4,432 eyes in 2,216 patients). The primary causes of mild visual impairment in patients with T2DM were cataract and DR [10]. In addition, we also identified 420 eyes of 210 patients with unexplained mild visual impairment (UMVI) in both eyes—21.3% of cases and 9.1% of all 2,216 participants. We believe that UMVI occurred because of the early function loss of the macular photoreceptor cells when no morphological changes could be detected in the population-based epidemiological studies in which fundus photography and optical coherence tomography (OCT) were the major detective techniques. Given that such a high proportion of patients with UMVI and the global increase in T2DM [11], the number of diabetic patients with UMVI is about to increase rapidly [5]. To date, no published study had addressed the pathogenesis of UMVI, while patients with UMVI continued to seek an explanation, because the characterization of the etiology as “unknown” would indicate a possible rapid progression to moderate or severe visual loss and imply that there is no effective prevention or treatment.

The mechanism of the ocular complications of T2DM is complex and still not well-demonstrated. Genetic susceptibility, inflammation, oxidative stress, and environmental influences were all reported to be involved [12–15]. Peroxisome proliferator-activated receptor gamma (PPARG) is a ligand-activated transcription factor that plays an important role in the control of a variety of physiological processes such as metabolism, angiogenesis, fibrosis, inflammation, and oxidative stress in various blind-causing diseases, such as DR, age-related macular degeneration, and optic neuropathy [16–19]. Genetic susceptibility determines the different responses to factors like inflammation. For example, the incidence of DR differed in different individuals with the same blood glucose level. Therefore, we speculated that the susceptibility of certain genes in the diabetic population may result in UMVI. In the present study, peroxisome proliferator-activated receptor gamma (PPARG) was chosen as a candidate gene, and we investigated whether the presence of PPARG gene polymorphisms was associated with UMVI in a Chinese Han T2DM population to provide novel insight into the pathogenesis of UMVI.

## 2. Materials and Methods

This was a population-based case-control study. The patients in the UMVI group and NV group were mainly diabetic residents in the Shanghai Xinjing community [9]. The study was approved by the Ethics Committee of the Shanghai general hospital, Shanghai Jiaotong University (2013KY023). All the procedures were conducted according to the tenets of the Declaration of Helsinki. Informed consent was obtained from all subjects after a full explanation of the study protocol.

## 3. Patient Selection

The inclusion criteria were (1) provision of the written informed consent, (2) diagnosis of T2DM based on the WHO diagnostic criteria, [20] (3) ability to comply with all the required examinations, (4) BCVA < 20/25 and ≥20/63 in both eyes, with no visual impairment-causing ophthalmic diseases, and (5) age- and gender-matched patients with BCVA ≥ 20/25 in both eyes [6].

The exclusion criteria were (1) eyelid diseases, strabismus, corneal diseases, lens diseases, and other eye diseases that may affect the results of OCTA or fundus photograph examinations; (2) eye diseases, such as glaucoma and macular degeneration, which may cause other fundus retinal microvasculopathy; (3) primary systemic diseases, including those involving the respiratory system, circulatory system, and urinary system in addition to DM; and (4) a history of cancer or major surgery.

The research team consisted of the same fully trained and experienced routine members as introduced before [9, 21]. First, the baseline characteristics were surveyed using a questionnaire. Patients met the above terms and went through a thorough eye examination; 1 ml of fasting whole peripheral blood was collected from each participant, and DNA extraction was performed according to the kit instructions (QIAN amp Blood kit Hilden, Germany).

## 4. SNPs Selection

Four PPARG single-nucleotide polymorphisms (SNPs) (rs1801282, rs3856806, rs709158, and rs10865710) in previous studies associated with metabolic disorders captured in the locus were selected [22–27]. Among them, rs1801282 is a confirmed type 2 diabetes susceptibility locus of PPARG [28]. rs3856806, rs709158, and rs10865710 are all associated with loci of lipoprotein metabolism and obesity in the Chinese Han population [25, 29, 30]. Probe sequences of four SNPs were shown in Table 1; positions and functional consequences were also listed.

## 5. Sequencing Methods

The sequences, which included both upstream and downstream regions of the target SNPs ([Supplementary-material supplementary-material-1]), were sent to Fluidigm (http://Assay_Design_Group@fluidigm.com), and the Fluidigm SNP genotyping markers which was composed of a specific target amplification (STA) primer, a locus-specific (LS) primer, and two allele-specific primers were designed. Genotyping was performed following the Fluidigm SNP genotyping instructions by the IRRI genotyping service laboratory (http://gsl@irri.org) as introduced by Kim et al. [31]. Briefly, the target region was amplified with the STA and LS primers under a thermal cycler. The diluted PCR products from the 268 samples, four Fluidigm SNP markers, and PCR reagents were simultaneously mated in a FR192.24 Dynamic Array by the IFC Controller. Then, PCR was performed in the FC1™ Cycler, and the fluorescence signals from the end PCR products were finally read under the EP1TM Reader.

## 6. Statistical Analysis

Student's *t*-test and the *χ*^2^ test were used to compare continuous clinical data and categorical variables, respectively. Allelic and genotypic frequencies between the UMVI and NV groups were compared by the *χ*^2^ test or Fisher's exact test. Hardy–Weinberg equilibrium (HWE) for genotype frequencies of the SNPs was tested by the *χ*2 test. The correction for multiple testing in the haplotype analysis was performed by permutation testing. Pairwise linkage disequilibrium (LD, D′) analyses between the polymorphisms and EM-based haplotype association analysis were performed by HAPLOVIEW (ver. 4.0) and SPSS 22.0 software (IBM Corporation, US). Odds ratios (OR) and 95% confidence intervals (CI) were also calculated. A *p* < 0.05 was considered statistically significant.

## 7. Results

A total of 135 T2DM residents with UMVI were admitted in the case group of this study. Another 133 normal vision (NV; BCVA ≥ 20/25 in both eyes) residents with T2DM were enrolled in the control group of this study.

Basic information for the subjects in the two groups is shown in Table 2. Except for the difference in the waistline and hipline, the two groups showed no statistically significant intergroup differences in gender, age, age at onset of T2DM, duration of diabetes, hemoglobin A1c levels, fasting blood glucose levels, and systolic and diastolic blood pressure. The UMVI group had a slightly shorter waistline and hipline than the NV group, but the well-acknowledged indicator of obesity degree, BMI, did not differ significantly between the two groups. Logistic regression analysis also did not reveal any correlation between the waistline, hipline, and target SNPs.

Out of the 4 SNPs selected, rs709158 had a low genotyping call rate (= 51%), while the remaining three had a full call rate. 4 SNPs tested in the UMVI and NV groups all qualified the HWE (*p* > 0.05). The allelic frequencies for each of the four sequence variants analyzed (rs3856806, rs1801282, rs709158, and rs10865710) in all the UMVI, and control cases are shown in Table 1.

The observed genotype frequencies of the 4 PPARG SNPs met the HWE (*p* > 0.05) in both the UMVI and NV groups (as shown in Table 3). Statistically significant differences were observed between the UMVI subjects and controls when the genotypic frequencies for each of the 3 SNPs with significantly increased allelic frequency (rs10865710, rs709158, and rs3856806) were compared. The frequency of genotype GC at SNP rs10865710 was significantly higher in the UMVI group (*p* < 0.001; GG + GC versus CC), conferring an approximately 8.94-fold increased risk for UMVI (OR = 8.94, 95% CI: 4.90–16.31), whereas genotype CC decreased the risks (OR = 0.07, 95% CI: 0.03–0.14). Genotype TT at SNP rs3856806 was strongly associated with UMVI (*p* < 0.0001, TT + TC versus CC) conferring a more than 3-fold increased risk (OR = 4.74, 95% CI: 2.68–8.54), whereas genotype CC appeared to be protective for UMVI (OR = 0.55, 95% CI: 0.37–0.82).

**Figure 1:**
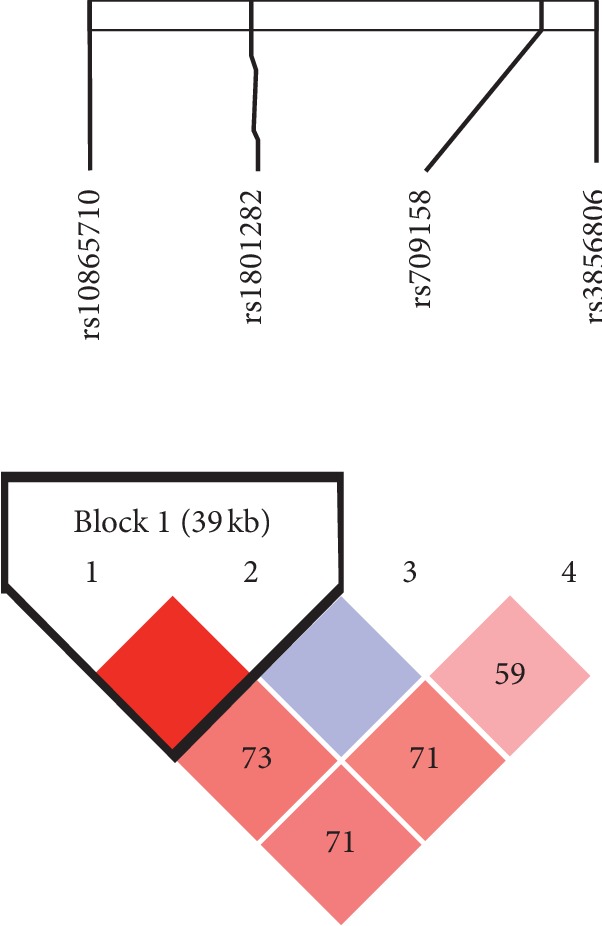
PPARG linkage disequilibrium plot of the PPARG single-nucleotide polymorphisms rs3856806, rs1801282, rs709158, and rs10865710. The number in the diamond refers to D0 (100 9 D0). The linkage disequilibrium block was defined according to the standard confidence intervals. The strength of linkage disequilibrium is depicted by the intensity of red coloring, which moves from white to light red as D0 progresses from 0 to 100.

The pairwise LD analysis identified one block (39 kb) (Figure 1), which included 4 SNPs in strong LD, as observed by the D0 value. The SNP rs10865710 was in complete LD with rs1801282 (coefficient of LD [D0] = 1.00). The frequency of these haplotypes and their associations with UMVI is shown in Table 4.

## 8. Discussion

A PubMed search indicated that there is no worldwide study that has investigated the association of PPARG gene polymorphisms with UMVI in a T2DM population. Therefore, the statistically significant relationship between UMVI and the SNPs rs10865710 and rs3856806 found in our study will be important for elucidating the gene susceptibility and possible pathogenesis of UMVI. Haplotype analysis for PPARG SNPs in the groups UMVI and NV is shown in Table 5.

Visual impairment in patients with T2DM is attributed primarily to retinal damage. [19, 32] The outer retina consists of photoreceptor neurons and Müller cells, which are metabolically coupled to support the generation of electrochemical impulses in response to stimulation of light, with nutrients and oxygen diffusing from choroidal vessels through the pigmented epithelium layer. The retina and choroid, as high-energy consumption targets, are highly prone to hyperglycemia-induced molecular damage. Quite a few published papers have confirmed that PPARG plays an important role in reactive oxygen species generation, inflammation, apoptosis, and antiangiogenesis-induced retinal and choroidal dysfunction [33–36]. Suppression of PPARG via activation of nuclear factor kappa B is reported to be involved in the pathogenesis of experimental DR and oxygen-induced retinopathy [37]. As an important constituent of mitochondrial reactive oxygen species imbalance, PPARG was also confirmed to be an initiated and sustained factor in the general pathways of DR after short-term stimulation by hyperglycemia and directly mediated the inhibitory effect of statins on reactive oxygen species, thus reducing early retinal injury in diabetic eyes [38, 39].

Specific variants rs10865710 (introns) and rs3856806 (synonymous mutation) (Table 1) account for UMVI, do not change the sequence of amino acids, and mainly affect the process of PPARG transcription. Therefore, we speculate that the differences in PPARG gene susceptibility lead to different levels of PPARG protein, which further result in differences in the response to oxidative stress in retina/choroid under the stimulus of hyperglycemia, causing early function loss of macular photoreceptor cells and eventually resulting in UMVI.

In that case, PPARG agonists, such as pioglitazone, may help control UMVI and relieve patient anxiety. Pioglitazone has been proven to protect retinal and/or choroidal cells from hyperglycemia-induced injuries in a PPARG-dependent pathway. It can normalize insulin signaling in the diabetic rat retina through reduction in the levels of tumor necrosis factor and suppressor of cytokine signaling 3, [35] modulate the retinal pigmented epithelium survival responses to oxidative stress, inhibit activation of the glial cells, prevent cell apoptosis, and protect the retina from subsequent cellular damage caused by retinal ischemia/reperfusion [33, 40]. In previously clinical studies, pioglitazone has been used to prevent vascular complications of T2DM, such as stroke and atherosclerosis [41, 42]. Therefore, we suppose that pioglitazone may be used to prevent visual impairment progression in patients with T2DM.

In summary, the present study confirmed an independent association between UMVI and PPARG polymorphisms in a T2DM population. The limitations of this study should not be neglected. First, the study was a single-center study based on the Chinese Han population and contained a small number of subjects. Second, more SNPs of PPARG should be sequenced. Further studies are also necessary.

## Figures and Tables

**Table 1:** Probe sequence for four SNPs used for Fluidigm sequencing analysis.

Rs number	SNP	Ch	Functional consequence	Position	SNP_SEQ
rs10865710	Intron C>G	3	Upstream transcript variant	12,311,699	AGTTTCATGTAGGTAAGACTGTGTAGAATGTCGGGTCTCGATGTTGGCGCTATTCAAGCCCTGATGATAAGGCTTTTGGCATTAGATGCTGTTTTGTCTT[C/G]ATGGAAAATACAGCTATTCTAGGATCCTTGAGCCTTTCATAAGAGATAAGGTTGTGAATCCTAAGACCCTAGGACCRTTTACTTAGATGATCTGCTCTCT

rs1801282	Intron C>G	3	Missense variant/coding sequence variant	12,351,626	TTGATCTTTTGCTAGATAGAGACAAAATATCAGTGTGAATTACAGCAAACCCCTATTCCATGCTGTTATGGGTGAAACTCTGGGAGATTCTCCTATTGAC[C/G]CAGAAAGCGATTCCTTCACTGATACACTGTCTGCAAACATATCACAAGGTAAAGTTCCTTCCAGATACGGCTATTGGGGACGTGGGGGCATTTATGTAAG

rs709158	Intron A>G	3	Genic downstream transcript variant	12,403,176	CTCTGCAGCAGGCAAAAGCTCTTTTTGTTAATTCAAAACAGTTTGGAATCCATTTCAGTTCTTCCTAAACCTCCAAGATACGGGGGAGGAAATTCACTGG[A/G]TTTTACAATATATTTTTCAAGGCAAATTGCCATCGCCGTCCTAATGACAGAGAAGCTGCCGATATCACTACAACGGCTGCAGATGGCAAGTCATCCAGCC

rs3856806	C1341T	3	3 prime UTR variant/synonymous variant	12,415,557	CCCTGGAGCTCCAGCTGAAGCTGAACCACCCTGAGTCCTCACAGCTGTTTGCCAAGCTGCTCCAGAAAATGACAGACCTCAGACAGATTGTCACGGAACA[C/T]GTGCAGCTACTGCAGGTGATCAAGAAGACGGAGACAGACATGAGTCTTCACCYGCTCCTGCAGGAGATCTACAAGGACTTGTACTAGCAGAGAGTCCTGA

Ch: chromosome.

**Table 2:** Demographic and clinical characteristics of 135 residents with unexplained mild visual impairment (UMVI) and 133 residents with normal vision (NV).

	UMVI residents	NV residents	*p* value^*∗*^
Gender (male)	53	60	0.39
Age (year)	65.34 ± 5.41	64.82 ± 8.45	0.59
Age at diabetes onset (years)	58.48 ± 10.76	57.51 ± 10.15	0.39
Duration of diabetes (years)	6.86 ± 5.21	7.30 ± 5.86	0.52
Hemoglobin A1c (%)	7.24 ± 1.46	7.09 ± 1.34	0.36
Fasting blood glucose (mmol/l)	7.21 ± 2.08	7.17 ± 1.88	0.87
Body mass index (kg/m^2^)	25.00 ± 3.62	25.75 ± 3.51	0.09
Waistline (cm)	85.68 ± 9.93	88.27 ± 9.19	0.03
Hipline (cm)	94.98 ± 9.84	97.03 ± 6.77	0.04
Systolic blood pressure (mmHg)	140.02 ± 20.84	143.77 ± 19.69	0.14
Diastolic blood pressure (mmHg)	81.03 ± 11.97	80.74 ± 11.35	0.84

^*∗*^Student's *t*-test and *χ*^2^ test.

**Table 3:** PPARG allele frequencies in the 135 residents with unexplained mild visual impairment (UMVI) and 133 residents with normal vision (NV).

SNP	Alleles	UMVI residents	NV residents	*p* value^*∗*^	MAF	OR (95%CI)
		number (%)	number (%)			
rs10865710	G	163 (60.4)	14 (5.3)	6.57*E* − 42	0.33	27.42 (15.18–49.51)
	C	107 (39.6)	252 (94.7)			
rs1801282	G	22 (8.1)	0 (0)	1.99*E* − 06	0.04	NA
	C	248 (91.9)	266 (100)			
rs709158	G	95 (58.6)	24 (21.4)	1.00*E* − 09	0.43	5.20 (3.00–9.00)
	A	67 (41.4)	88 (78.6)			
rs3856806	T	88 (32.6)	21 (7.9)	1.22*E* − 12	0.20	5.64 (3.38–9.42)
	C	182 (67.4)	245 (92.1)			

MAF = minor allele frequency; OR = odds ratio; CI = confidence interval; NA, the odds ratio was not available where the number of individuals with two copies of the risk allele was zero. ^*∗*^*χ*^2^ test.

**Table 4:** Genotypic association analysis in 135 residents with unexplained mild visual impairment (UMVI) and 133 residents with normal vision (NV).

SNP	Genotype	UMVI residents	NV residents	HWpval	*p* value^*∗*^	OR (95% CI)^*∗*^	*p* value^#^	OR^#^ (95% CI)
		number	number					
rs10865710	GG	36	0	0.08	7.17*E* − 42	NA		
	GC	91	14			6.40 (3.47–11.80)	1.86*E* − 41	0.01 (0.00–0.02)
	CC	8	119			0.07 (0.03–0.14)	(CC: GG + GC)	
rs1801282	GG	0	0	1	NA	NA		
	GC	22	0			NA	7.46*E* − 6	0.46 (0.40, 0.53)
	CC	113	133			0.86 (0.61–1.21)	(CC: GG + GC)	
rs709158	GG	25	4	0.33	3.35*E* − 09	4.43 (1.46–13.43)	0.41	2.22 (0.67, 7.38)
	GA	45	16			1.94 (1.00–3.78)	6.9*E* − 7	20.46 (5.84, 71.61)
	AA	11	36			0.21 (0.10–0.45)	1.39*E* − 6	0.11 (0.04, 0.26)
rs3856806	TT	16	0	0.10	1.24*E* − 10	NA		
	TC	56	21			2.63 (1.51–4.58)	8.92*E* − 10	0.16 (0.09–0.29)
	CC	63	112			0.55 (0.37–0.82)	(CC: TT + TC)	

*p* value^*∗*^ (chi-square test); OR (95% CI) ^*∗*^(chi-square test); OR^#^ (95 % CI); *p* value^#^ (Bonferroni correction); OR' (Bonferroni correction); HWpval, Hardy–Weinberg equilibrium *p* value. OR = odds ratio; CI = confidence interval; NA, the odds ratio was not available where the number of individuals with two copies of the risk allele was zero. ^*∗*^*χ*^2^ test.

**Table 5:** Haplotype analysis for PPARG SNPs in 135 residents with unexplained mild visual impairment (UMVI) and 133 residents with normal vision (NV).

rs10865710	rs1801282	UMVI residents	NV residents	*p* value^*∗*^	OR (95% CI)
		haplotype frequency	haplotype frequency		
C	C	0.34	0.95	6.57*E* − 42	0.04 (0.02–0.07)
G	C	0.52	0.05	4.04*E* − 33	19.67 (10.92–35.45)
G	G	0.08	0	1.99*E* − 06	NA

^*∗*^ *χ* ^2^ test.

## Data Availability

The data used to support the findings of this study are included within the article.

## Supplementary Materials

Supplementary Materials Fluidigm SNP genotyping markers.
