# Supplementary material for: PPARG Polymorphisms Are Associated with Unexplained Mild Vision Loss in Patients with Type 2 Diabetes Mellitus
Source: J Ophthalmol. 2019 Dec 12;2019:5284867. doi: 10.1155/2019/5284867 (PMC6930731; doi:10.1155/2019/5284867)
Supplement: Supplementary Materials — Fluidigm SNP genotyping markers. [file 5284867.f1.pdf]

Supplement 1: Fluidigm SNP genotyping markers

	ALLELE	SNP_SEQ	ASP1_SEQ	ASP2_SEQ	LSP_SEQ	STA_SEQ	AMP_GC
rs10865710	C G	AGTTTCATGTAGGTAAGACTGTGTAGAATGTCGGGTCTCGATGTTGGCGCTATTCAAGCCCTGATGATAAGGCTTTTGGCATTAGATGCTGTTTTGTCTT[C/G]ATGGAAAATACAGCTATTCTAGGATCCTTGAGCCTTTCATAAGAGATAAGGTTGTGAATCCTAAGACCCTAGGACCRTTTACTTAGATGATCTGCTCTCT	TGGCATTAGATGCTGTTTTGTCTTC	TGGCATTAGATGCTGTTTTGTCTTG	GGCTCAAGGATCCTAGAATAGCTGT	CAAGCCCTGATGATAAGGCTTT	0.41
rs1801282	C G	TTGATCTTTTGCTAGATAGAGACAAAATATCAGTGTGAATTACAGCAAACCCCTATTCCATGCTGTTATGGGTGAAACTCTGGGAGATTCTCCTATTGAC[C/G]CAGAAAGCGATTCCTTCACTGATACACTGTCTGCAAACATATCACAAGGTAAAGTTCCTTCCAGATACGGCTATTGGGGACGTGGGGGCATTTATGTAAG	CAGTGAAGGAATCGCTTTCTGG	CAGTGAAGGAATCGCTTTCTGC	GCAAACCCCTATTCCATGCTGT	TGTGATATGTTTGCAGACAGTGTATC	0.47
rs3856806	C T	CCCTGGAGCTCCAGCTGAAGCTGAACCACCCTGAGTCCTCACAGCTGTTTGCCAAGCTGCTCCAGAAAATGACAGACCTCAGACAGATTGTCACGGAACA[C/T]GTGCAGCTACTGCAGGTGATCAAGAAGACGGAGACAGACATGAGTCTTCACCYGCTCCTGCAGGAGATCTACAAGGACTTGTACTAGCAGAGAGTCCTGA	TCAGACAGATTGTCACGGAACAC	TCAGACAGATTGTCACGGAACAT	TCCGTCTTCTTGATCACCTGCA	GCTGCTCCAGAAAATGACAGA	0.51
rs709158	A G	CTCTGCAGCAGGCAAAAGCTCTTTTTGTTAATTCAAAACAGTTTGGAATCCATTTCAGTTCTTCCTAAACCTCCAAGATACGGGGGAGGAAATTCACTGG[A/G]TTTTACAATATATTTTTCAAGGCAAATTGCCATCGCCGTCCTAATGACAGAGAAGCTGCCGATATCACTACAACGGCTGCAGATGGCAAGTCATCCAGCC	ATGGCAATTTGCCTTGAAAAATATATTGTAAAAT	TGGCAATTTGCCTTGAAAAATATATTGTAAAAC	CTCCAAGATACGGGGGAGGA	TCTGTCATTAGGACGGCGA	0.38

STA =specific target amplification， LSP= locus-specific primer，ASP=allele-specific primers，
